# Diagnostic value and prognostic significance of microRNA-210, serum creatinine, neutrophil gelatinase-associated lipocalin, blood urea nitrogen, cystatin C, and sequential organ failure assessment scores in patients with sepsis-associated acute kidney injury

**DOI:** 10.3389/fmed.2025.1671599

**Published:** 2026-01-06

**Authors:** Zhikun Zhang, Zhaolong Zhang, Jian Liu, Lujun Qiao, Xiaoguang Fan

**Affiliations:** Department of Intensive Care Unit, Shengli Oilfield Central Hospital, Dongying, Shandong, China

**Keywords:** blood urea nitrogen, cystatin C, microRNA-210, neutrophil gelatinase-associated lipocalin, sepsis-associated acute kidney injury, sequential organ failure assessment scores, serum creatinine

## Abstract

**Background:**

Sepsis-associated acute kidney injury is common in critically ill patients and is strongly associated with adverse outcomes.

**Objective:**

To explore the diagnostic value and prognostic significance of microRNA (miRNA) miR-210, serum creatinine (Scr), neutrophil gelatinase-associated lipocalin (NGAL), blood urea nitrogen (BUN), cystatin C (CysC), and sequential organ failure assessment (SOFA) scores in patients with sepsis-associated acute kidney injury.

**Design:**

This was a retrospective study.

**Setting:**

This study was performed in the Department of Intensive Care Unit at Shengli Oilfield Central Hospital.

**Participants:**

A total of 81 sepsis patients treated at our hospital from April to November 2021 were chosen and divided into two groups according to whether acute kidney injury was present.

**Interventions:**

The levels of miR-210, Scr, NGAL, BUN, CysC, and SOFA scores were detected, and an ROC curve was implemented to assess the diagnostic value. The risk factors influencing prognosis were assessed by binary logistic regression.

**Primary outcome measures:**

(1) Levels of miR-210, Scr, NGAL, BUN, CysC, and SOFA scores; (2) Diagnostic value of miR-210, Scr, NGAL, BUN, CysC, and SOFA scores; (3) Risk factors influencing the prognosis of patients with sepsis-associated acute kidney injury.

**Results:**

Compared with sepsis patients, levels of miR-210, Scr, NGAL, BUN, CysC, and SOFA scores in patients with sepsis-associated acute kidney injury were elevated (*p* < 0.001). The ROC curve indicated that miR-210 had the highest diagnostic value in patients with sepsis-associated acute kidney injury, with an AUC of 0.913 and sensitivity and specificity of 92.21 and 91.37%, respectively (*p* < 0.001). The expressions of miR-210, NGAL, CysC, and SOFA scores in the dead patients were significantly elevated compared to the survival cases (*p* < 0.05). MiR-210 levels and SOFA scores affected the prognosis of patients with sepsis-associated acute kidney injury.

**Conclusion:**

Our study demonstrated that miR-210, Scr, NGAL, BUN, CysC, and SOFA scores had significant diagnostic value in patients with sepsis-associated acute kidney injury and that miR-210 and SOFA scores had good prognostic value.

## 1 Introduction

The incidence of sepsis is high, with over 18 million cases of sepsis worldwide each year and 750,000 cases in the United States each year. This number is increasing at a rate of 1.5 to 8.0% per year (1). Sepsis is a dangerous condition with a high fatality rate, resulting in approximately 14,000 deaths per day worldwide and 215,000 deaths per year in the United States (2). Sepsis is a type of systemic inflammatory syndrome caused by the invasion of bacteria and other pathogenic microorganisms, and its pathogenesis is complex, including the direct damage of pathogenic microorganisms to tissues and organs and the disorder of immune function (3). Despite recent advances in anti-infective therapy and multi-organ functional support, the mortality rate of sepsis remains high (4). During sepsis, a combination of factors can induce kidney cell damage, including changes in renal perfusion that lead to cell hypoxia and the release of various mediators that alter cell metabolism (5). Sepsis-associated acute kidney injury is chronic and progressive damage to the kidney, resulting in renal atrophy, metabolic product retention, acid–base balance, and water and electrolyte balance disorders of the chronic syndrome and is one of the causes of a variety of infectious diseases (6). To further study the pathogenesis of sepsis-associated acute kidney injury and new methods of diagnosis and prognosis prediction, more and more scientists are beginning to apply gene regulation technology to the research of sepsis-associated acute kidney injury, and the search for molecular biological targets of sepsis-associated acute kidney injury has gradually become a research hotspot (7).

Blood urea nitrogen (BUN) is an indicator to judge whether renal function is damaged or not and the degree of damage (8). Serum creatinine (Scr) is a traditional indicator for clinical diagnosis of acute kidney injury, but due to low sensitivity and susceptibility to other factors, it is often difficult to meet the requirements of early clinical diagnosis (9). Cystatin C (CysC) belongs to a part of the cysteine protease inhibitor family, which is produced by the synthesis and release of nucleated cells (10). CysC is currently an ideal laboratory index for reflecting glomerular filtration rate in clinical practice. Most CysC can be removed from the body only after filtration through the glomerular filtration membrane, and it is not affected by age, gender, diet structure, or other factors of patients, so it can effectively reflect the abnormal situation of renal function (11). Neutrophil gelatinase-associated lipid carrier protein (NGAL), a type of lipocalin, is a small molecular weight secreted protein originally discovered in activated neutrophils (12). Modern studies have shown that NGAL is one of the most effective biological markers for the diagnosis of acute kidney injury, as well as one of the effective markers for early diabetic nephropathy (13, 14).

The sequential organ failure assessment (SOFA) score is an index system used to comprehensively evaluate the severity of multi-organ dysfunction in patients. It covers the functional status of multiple organ systems, including the respiratory system, coagulation system, liver, cardiovascular system, central nervous system, and kidneys (15). Among them, the coagulation function section is an important component of the SOFA score, primarily reflecting abnormal conditions of the patient’s coagulation function through indicators such as platelet count (16). Abnormal SOFA score for coagulation is a common complication in patients with sepsis-associated acute kidney injury. It may result in increased risk of bleeding, which may further exacerbate circulatory failure, leading to multiple organ failure and death (17).

**Figure 1:**
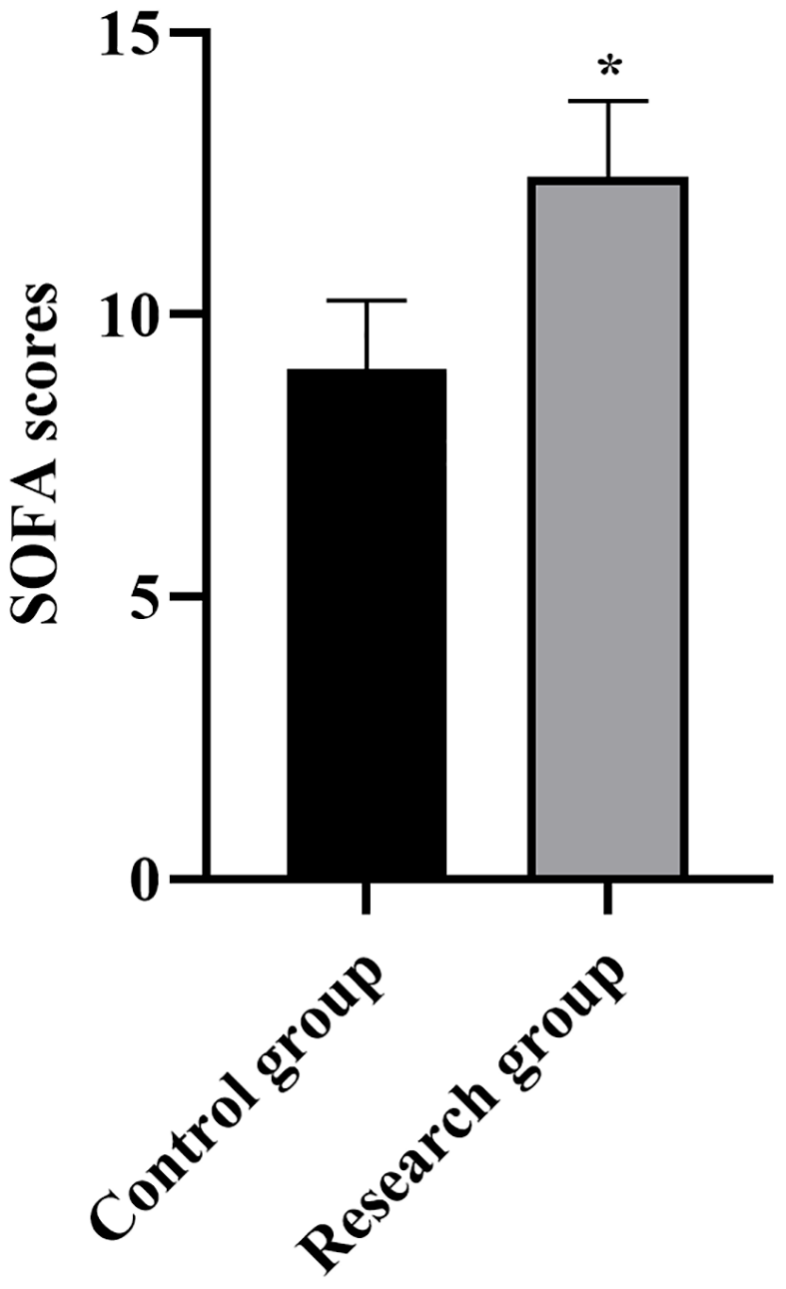
SOFA scores in both groups. *p < 0.05, compared with the control group. SOFA: Sequential Organ Failure Assessment.

**Figure 2:**
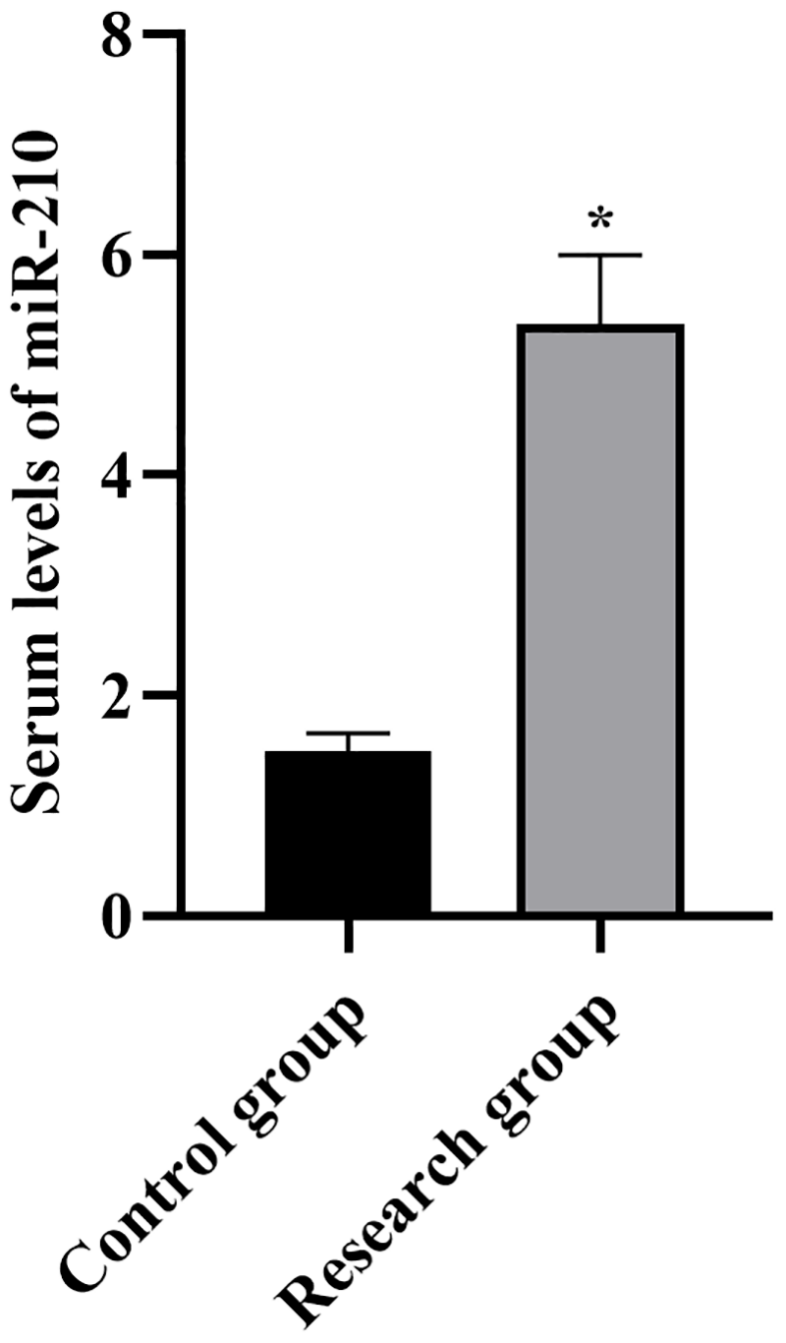
Serum levels of miR-210 in both groups. *p < 0.05, compared with the control group.

**Figure 3:**
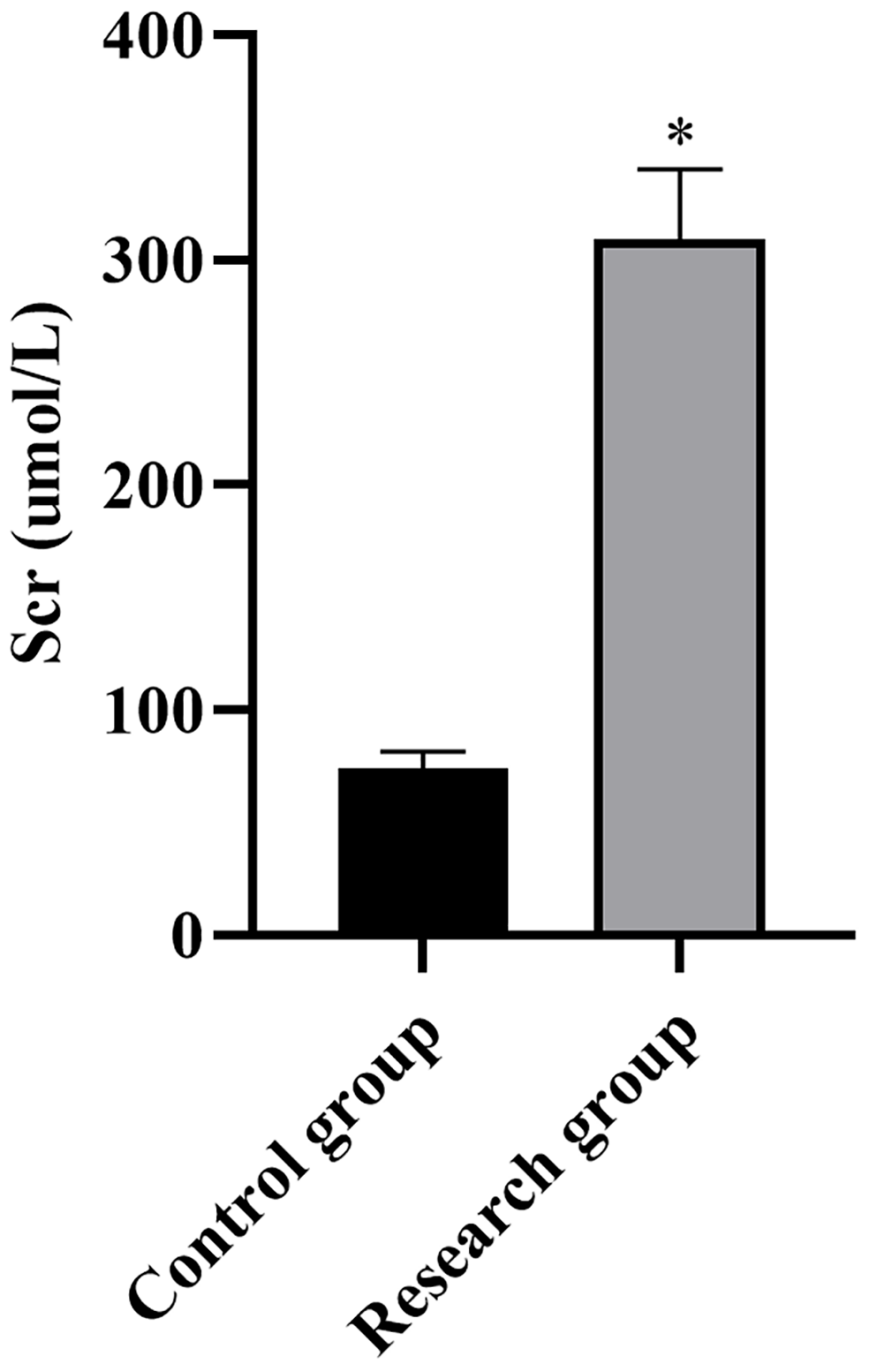
Scr levels in both groups. *p < 0.05, compared with the control group. Scr: serum creatinine.

**Figure 4:**
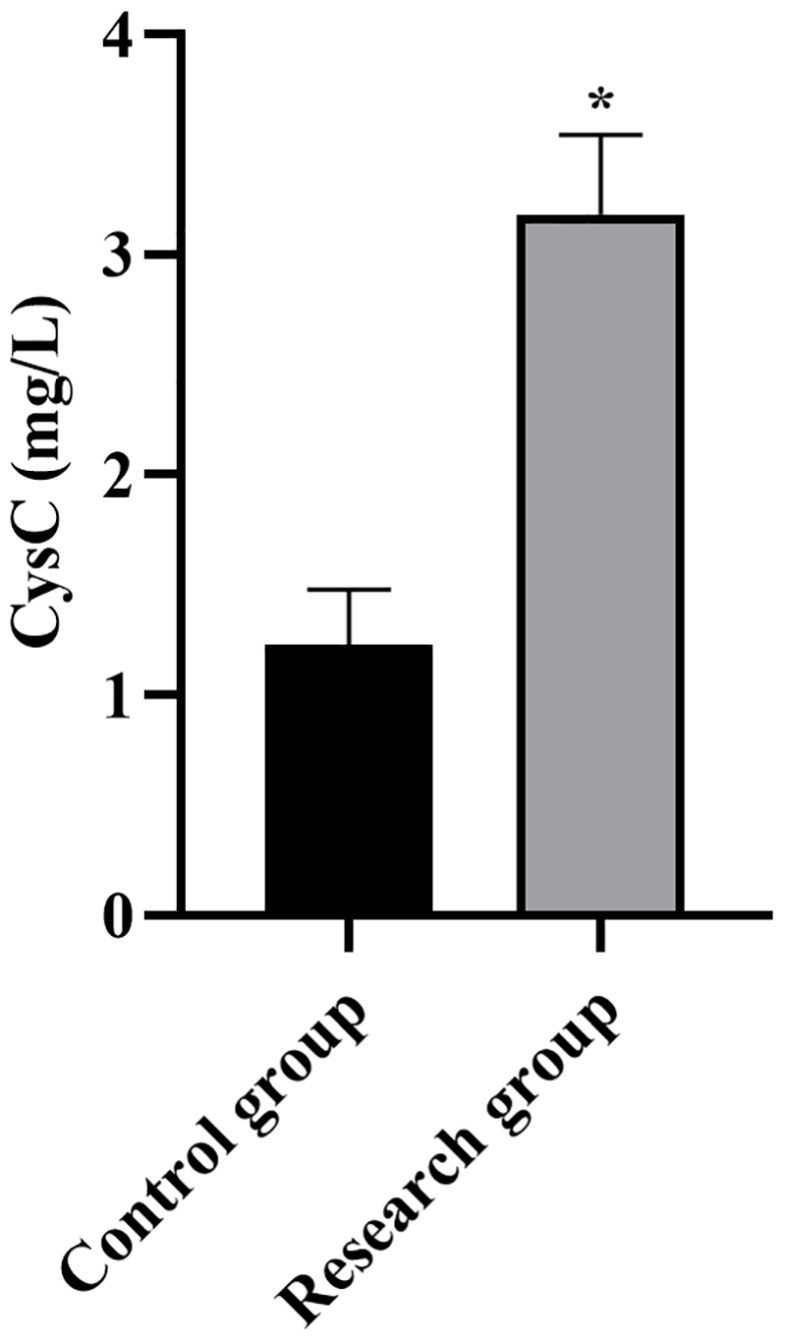
Serum CysC levels in both groups. *p < 0.05, compared with the control group. CysC: cystatin C.

**Figure 5:**
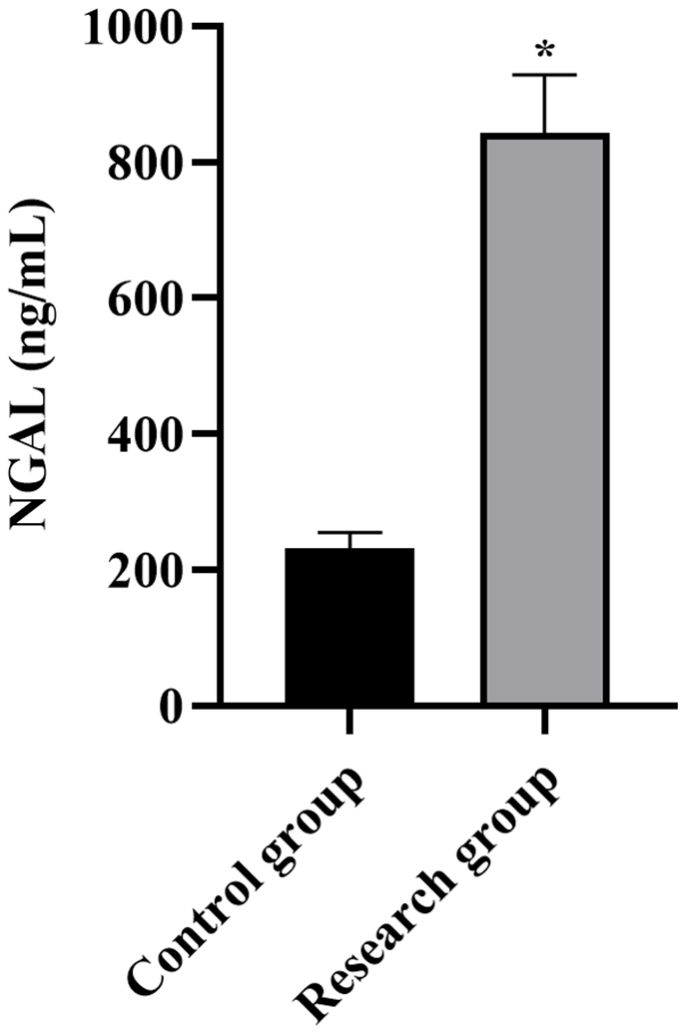
Serum NGAL levels in both groups. *p < 0.05, compared with the control group. NGAL: neutrophil gelatinase-associated lipocalin.

**Figure 6:**
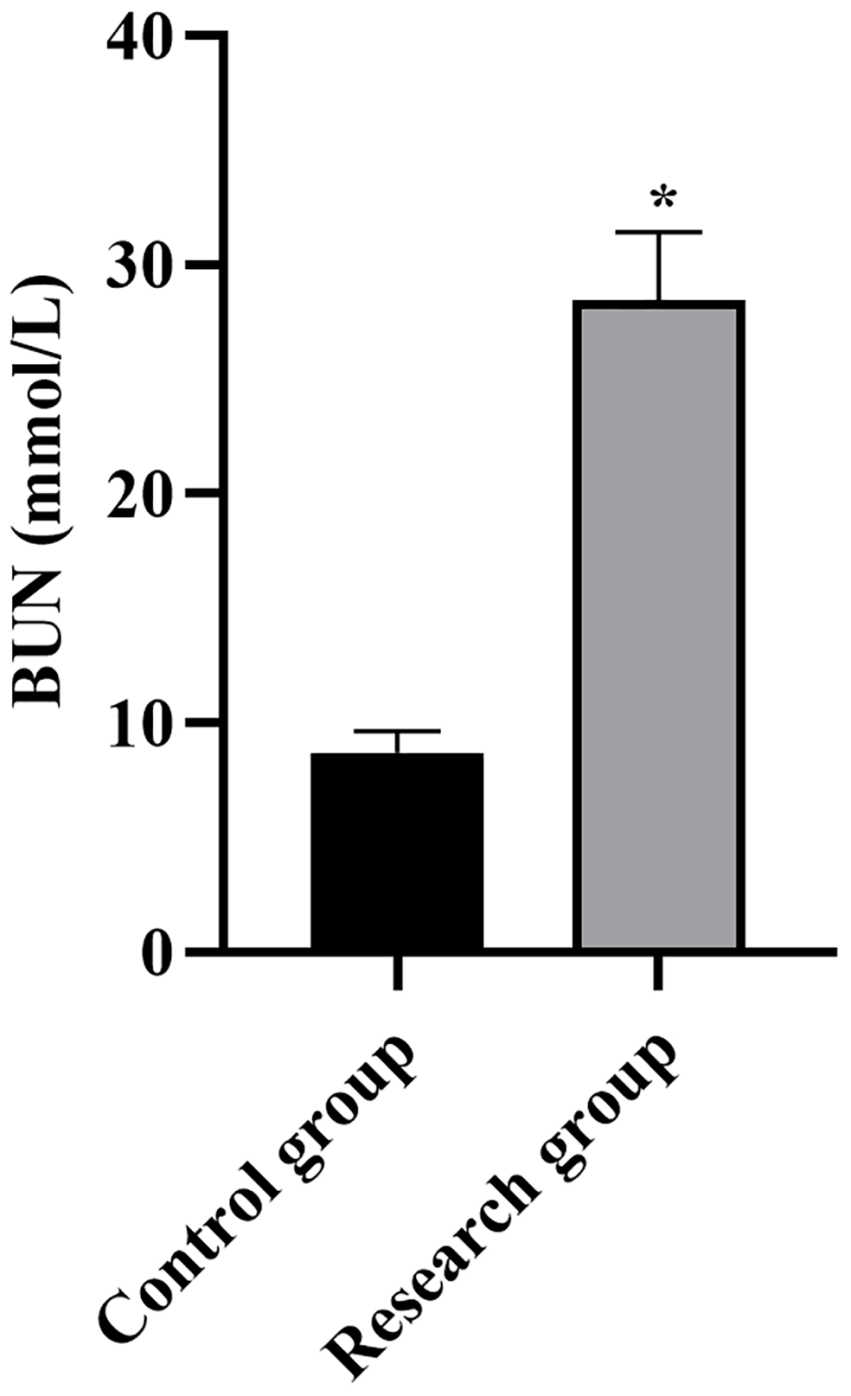
Serum BUN levels in both groups. *p < 0.05, compared with the control group. BUN: blood urea nitrogen.

Recent literature has demonstrated that microRNAs (miRNAs) play a modulatory role in biological processes such as cell proliferation, apoptosis, immune response, and inflammation, and are closely linked to the progression of kidney diseases. (18) As reported previously, exosomal miR-30d-5p of neutrophils induces M1 macrophage polarization and primes macrophage pyroptosis in sepsis-related acute lung injury (19). MiRNA-133a aggravates inflammatory responses in sepsis by targeting SIRT1 (20). More importantly, miR-210 was reported to be upregulated in patients with sepsis-induced acute kidney injury and to predict the prognosis and survival of patients with sepsis-induced acute kidney injury (21). From the perspective of biological mechanisms, miR-210 is a type of miRNA that is induced by hypoxia. Its expression is usually precisely regulated by hypoxia-inducible factor-1α (HIF-1α). During the occurrence and development of sepsis, the body is often in a severe hypoxic state, and this hypoxic environment will activate HIF-1α. Activated HIF-1α can specifically bind to the promoter region of the miR-210 gene, thereby initiating the transcription process of miR-210 and significantly increasing its expression level (22). The upregulated miR-210 plays multiple crucial roles within the cells and is closely associated with physiological and pathological processes such as tissue hypoxia, mitochondrial stress, and metabolic reprogramming. In terms of tissue hypoxia, miR-210 can promote the formation of new blood vessels by regulating the expression of a series of genes related to angiogenesis, attempting to alleviate the hypoxic condition of the tissue (23). At the mitochondrial stress level, the systemic inflammatory response triggered by sepsis can cause severe damage to mitochondria, leading to mitochondrial dysfunction and subsequently affecting the energy metabolism and normal physiological functions of cells (24). miR-210 can alleviate the oxidative stress damage to mitochondria by regulating genes related to mitochondrial function, stabilize the mitochondrial membrane potential, maintain the normal morphology and function of mitochondria, and enhance the cell’s tolerance to hypoxia and inflammatory damage (25). From the perspective of metabolic reprogramming, under hypoxic conditions, cells need to undergo a metabolic transformation to adapt to the harsh survival environment. miR-210 can participate in regulating the processes of cellular glucose metabolism and lipid metabolism, promoting the transformation of cells from aerobic metabolism to anaerobic glycolysis, providing necessary energy support for the cells, and regulating lipid synthesis and breakdown to maintain the balance of metabolism within the cells (26). However, in sepsis-associated acute kidney injury, the value of miR-210 in the combined application with other biomarkers for diagnosis and prognosis assessment still requires further in-depth exploration (Figures 1–6).

This study aimed to further verify the diagnostic and prognostic value of miR-210 in patients with sepsis-induced acute kidney injury at a single-center intensive care unit in China. At the same time, we comprehensively analyzed the diagnostic efficacy and prognostic predictive ability of miR-210, Scr, NGAL, BUN, CysC, and SOFA score in sepsis-induced acute kidney injury. The expectation is to provide a more comprehensive and accurate basis for the early diagnosis and prognosis assessment of sepsis-induced acute kidney injury.

## 2 Data and methods

### 2.1 Clinical data

A total of 81 patients with sepsis who were treated in our hospital from April to November 2021 were chosen as the research objects. They are divided into two groups according to whether acute kidney injury was combined. Among them, 41 patients with acute kidney injury were included in the research group, and 40 patients without acute kidney injury were included in the control group. In the research group, 27 males together with 14 females were included, aged from 30 to 88 years old, with a mean age of 67.56 ± 13.47 years. In the control group, 25 males and 15 females were included, aged 29 to 90 years old, with a mean age of 64.42 ± 13.58 years. No significant differences in gender or age were observed between the two groups (*p* > 0.05).

To more accurately analyze the impact of various indicators on the prognosis of patients with sepsis-associated acute kidney injury, we specifically defined the time point for the 28-day mortality rate result. Specifically, starting from the day of the patient’s admission as Day 0, the follow-up continued until Day 28. During this period, the patient’s survival status was meticulously recorded through daily ward visits, regular telephone follow-ups, and review of the hospital’s electronic medical record system. If the patient died for any reason before Day 28 (including on the 28th day itself), it was classified as a death event; if the patient survived until Day 28 or remained alive on Day 28, it was classified as a survival event. Through this clear definition of time and rigorous follow-up method, the accuracy and reliability of the 28-day mortality rate results were ensured. In addition, patients with sepsis-associated acute kidney injury were divided into a survival group (18 cases) and a death group (23 cases) according to 28-day outcome.

### 2.2 Inclusion and exclusion criteria

Inclusion criteria: (1) Patients met relevant diagnostic criteria for sepsis and/or acute kidney injury, specifically: The criteria for diagnosing sepsis are based on the standards established by the Third International Consensus Conference on Sepsis-3 in 2016. For patients suspected of having an infection, the presence of infection is determined through a comprehensive assessment of clinical manifestations and pathogen detection results. Clinical manifestations include fever, chills, abnormal white blood cell count, and other common signs of infection; pathogen detection includes blood culture, sputum culture, urine culture, and other microbial cultures, as well as polymerase chain reaction (PCR) for detecting pathogen nucleic acids. A positive result from any of these tests, combined with clinical manifestations, can confirm the presence of infection. Based on this, the SOFA score is calculated. If the score increases by ≥ 2 points compared to the baseline, sepsis is diagnosed. The diagnostic criteria for sepsis-associated acute kidney injury are as follows: when sepsis is diagnosed, the diagnosis is made according to the KDIGO acute kidney injury standards. Any of the following conditions being met is sufficient: (a) Scr indicator: An increase of ≥0.3 mg/dL (26.5 μmol/L) in Scr within 24 h or a rise to 1.5 times or more of the baseline value, and the increase is clearly or inferentially observed to have occurred within the previous 7 days. (b) Urine volume indicator: Urine volume <0.5 mL/(kg·h) for more than 6 h. At the same time, other causes of reduced urine volume, such as dehydration, urinary tract obstruction, and prerenal azotemia, should be excluded. These can be differentiated through a detailed collection of medical history, a comprehensive physical examination, and imaging examinations such as a urinary system ultrasound. (2) Patients were older than 18 years old. (3) All patients and their families signed to participate in this study.

Exclusion criteria: (1) Patients with malignant tumor disease or autoimmune disease. (2) Patients who had been clearly diagnosed with acute kidney diseases (such as acute glomerulonephritis, etc., and whose kidney function has not been impaired after treatment) or chronic kidney diseases (including chronic glomerulonephritis, diabetic nephropathy, and hypertensive nephropathy at various stages of chronic kidney disease, with underlying abnormal kidney function). (3) Pregnant or lactating women.

### 2.3 Detection of serum markers

To ensure the accuracy and consistency of the research data, blood samples from all the enrolled patients were collected within 24 h after admission. After the patient was admitted to the hospital, a professional nurse collected 5 mL of venous blood using a vacuum blood collection tube containing an anticoagulant under strict aseptic conditions. The samples were centrifuged at 3,000 r/min for 10 min with a 15 cm radius. The supernatant was kept at −80 °C for examination. Scr level was detected by an automatic biochemical analyzer. Serum NGAL was detected by double-antibody sandwich enzyme-conjugate immunoassay (kit provided by Shanghai Bangjing Industrial Co., LTD., batch No.: 20161124). BUN levels were detected by enzyme-linked immunosorbent assay (Shanghai Fanwei Biotechnology Co., LTD). The serum Cys-C level was detected by immunoturbidimetry.

### 2.4 Detection of serum miR-210

MiR-210 expression in serum was detected by real-time quantitative fluorescence polymerase chain reaction (PCR). In both groups, 5 mL of peripheral blood samples were collected immediately after admission to the ICU, serum miRNAs were extracted using a miRNA test kit (purchased from Invitrogen in the United States), and then reverse transcribed into cDNA strands using a reverse transcription kit (purchased from Fermentas Company in the United States), and the specific operation procedure was carried out according to the instructions of the test kit. The internal reference was small nuclear RNA U6 (snRNA U6). The PCR reaction system and conditions were determined according to the kit instructions: predenaturation at 95 °C, 5 min, 95 °C, 10 s denaturation, 60 °C, 20 s annealing, 72 °C, 20 s extension, 40 cycles. The expression level of miR-210 was calculated by the 2^−ΔΔCt^ method. In this study, we used the “control group (without acute kidney injury)” as the calibration group to calculate the 2^−ΔΔCt^ value. Specifically, we first obtained Ct values for miR-210 and the reference U6 in the research and control groups through using RT-qPCR. We then calculated the ΔCt values for miR-210 relative to U6 in the research and control groups (ΔCt = Ct_miR-210 − Ct_U6). Finally, using the ΔCt value of the control group as the calibration, we calculated the ΔΔCt value of the research group (ΔΔCt = ΔCt_research group − ΔCt_control group). We then expressed the relative expression level of miR-210 in the research group using 2^−ΔΔCt^. The primer sequences of miR-210 and U6 were as follows: miR-210: F: 5′-CTGGAGCTGTGCGTGTGACAGC-3′, R: 5′-GTGCAGGGTCCGAGGT-3′. U6: F: 5′-AGAGAAGATTA GCATGGCCCCTG-3′, R: 5′-ATCCAGTGCAGGGTCCGAGG-3′.

### 2.5 Sepsis-related organ failure assessment (SOFA)

The SOFA score is used to assess the degree of organ dysfunction in patients upon admission (27). The calculation time point was set within the first 24 h after the patient’s admission. The SOFA score consisted of six parts, including respiratory (PaO_2_/FiO_2_, respiratory support), coagulation system (platelets), liver function (bilirubin), circulatory system (mean arterial pressure, dopamine, epinephrine, norepinephrine, dobutamine dose), nervous system score, and renal system (creatinine, 24-h urine output). Scores ranged from 0 to 4 for each group, with the total score ranging from 0 to 24. A higher score represented a worse prognosis. The SOFA score was assessed by two associate chief physicians of the ICU specialty.

### 2.6 Statistical analysis

Statistical software SPSS 22.0 (IBM Corp., Armonk, NY, USA) was implemented to analyze the data. For continuous variables, the Shapiro–Wilk test was used to assess normality. If the data follow a normal distribution, the measurement data are presented in the form of (*x* ± *s*) and are compared using the independent samples *t*-test; if the data do not follow a normal distribution, the measurement data are presented in the form of the median (interquartile range) [M (Q1 − Q3)]. They are compared using the Mann–Whitney *U* test. Statistical data were expressed as (*n*, %), and the *χ*^2^ test was used for comparison. The ROC method was implemented to evaluate the predictive value of miR-210, Scr, NGAL, BUN, CysC, and SOFA scores in patients with sepsis-associated acute kidney injury. When conducting the ROC curve analysis, the synchrony between increases in miR-210 levels and the increase in Scr levels was fully considered. The time point at which the Scr level reached the diagnostic criteria for acute kidney injury was set as the diagnostic benchmark. The diagnostic value of each indicator detected within 24 h of admission for predicting the time point at which the Scr level met the diagnostic criteria for acute kidney injury was analyzed, and the role of these indicators was evaluated based on the observed temporal correlation. The area under the receiver operating curve (AUC) represented the diagnostic value (the diagnostic value was low when AUC was between 0.5 and 0.7, the diagnostic value was moderate when AUC was between 0.7 and 0.9, and the diagnostic value was high when AUC was above 0.9). The risk factors influencing prognosis were analyzed by binary logistic regression. *p* < 0.05 was considered statistically significant.

## 3 Results

### 3.1 Serum levels of miR-210, Scr, NGAL, BUN, CysC, and SOFA scores between the two groups

Compared with the control group, the levels of miR-210, Scr, NGAL, BUN, CysC, and SOFA scores in the research group were higher (*p* < 0.001, Table 1).

**Table 1:** Serum levels of miR-210, Scr, NGAL, BUN, CysC, and SOFA scores between the two groups.

Indexes	Control group (*n* = 40)	Research group (*n* = 41)	*t*-value	*p*-value
miR-210	1.50 ± 0.16	5.37 ± 0.63	37.67	*p* < 0.001
Scr (μmol)	73.99 ± 7.42	309.01 ± 31.28	46.25	*p* < 0.001
NGAL (ng/mL)	231.79 ± 23.25	843.29 ± 85.24	43.80	*p* < 0.001
BUN (mmol/L)	8.73 ± 0.91	28.46 ± 2.96	40.33	*p* < 0.001
CysC (mg/L)	1.23 ± 0.25	3.18 ± 0.36	28.24	*p* < 0.001
SOFA scores (points)	9.03 ± 1.23	12.44 ± 1.36	11.82	*p* < 0.001

### 3.2 Diagnostic value of miR-210, Scr, NGAL, BUN, CysC, and SOFA scores in patients with sepsis-associated acute kidney injury

The ROC curve indicated that miR-210 had the highest diagnostic value in patients with sepsis-associated acute kidney injury, with an AUC of 0.913, sensitivity and specificity of 92.21 and 91.37%, respectively, as shown in Table 2 (*p* < 0.001).

**Table 2:** Diagnostic value of miR-210, Scr, NGAL, BUN, CysC, and SOFA scores in patients with sepsis-associated acute kidney injury.

Indexes	95% CI	AUC	*p*-value	Sensitivity	Specificity	Cut-off values
miR-210	0.846–0.978	0.913	<0.001	92.21%	91.37%	3.06
Scr (μmol)	0.601–0.817	0.715	<0.001	71.02%	70.29%	232.36
NGAL (ng/mL)	0.723–0.912	0.817	<0.001	83.84%	79.56%	438.67
BUN (mmol/L)	0.603–0.821	0.717	<0.001	72.23%	69.58%	18.69
CysC (mg/L)	0.623–0.856	0.742	<0.001	75.42%	76.58%	2.04
SOFA scores (points)	0.614–0.836	0.723	<0.001	73.47%	72.19%	12.37

Scr, serum creatinine; NGAL, neutrophil gelatinase-associated lipocalin; BUN, blood urea nitrogen; CysC, cystatin C; SOFA, sequential organ failure assessment.

### 3.3 Univariate analysis of influence on prognosis of sepsis-associated acute kidney injury

As shown in Table 3, serum levels of miR-210, NGAL, CysC, and SOFA scores were significantly higher in the death group than in the survival group, whereas no significant differences were observed in age, sex, serum creatinine, or blood urea nitrogen. No significant difference was found in age, gender, Scr, and BUN between the groups (*p* > 0.05).

**Table 3:** Univariate analysis of influence on prognosis of sepsis-associated acute kidney injury.

Variables	Survival	Dead	*p*-value
Cases	18	23	
Age (years)	67.43 ± 12.58	67.58 ± 12.43	NS
Gender (male/female)	12/6	15/8	NS
miR-210	4.35 ± 0.53	5.41 ± 0.63	<0.05
Scr (μmol)	288.46 ± 31.02	335.26 ± 34.17	NS
NGAL (ng/mL)	711.06 ± 72.34	935.58 ± 94.29	<0.05
BUN (mmol/L)	27.15 ± 2.86	30.13 ± 3.25	NS
CysC (mg/L)	2.91 ± 0.33	3.54 ± 0.42	<0.05
SOFA scores (points)	11.44 ± 1.23	13.22 ± 1.45	<0.05

Scr, serum creatinine; NGAL, neutrophil gelatinase-associated lipocalin; BUN, blood urea nitrogen; CysC, cystatin C; SOFA, sequential organ failure assessment; NS, no significance.

### 3.4 Prognostic risk factors for patients with sepsis-associated acute kidney injury by logistic regression analysis

The statistically significant miR-210, NGAL, CysC, and SOFA scores in the univariate analysis were substituted into the Logistic regression model. The results uncovered that miR-210 expression and SOFA scores affected the prognosis of patients with sepsis-associated acute kidney injury (*p* = 0.02 and *p* = 0.03, Table 4).

**Table 4:** Prognostic risk factors for patients with sepsis-associated acute kidney injury by logistic regression analysis.

Characteristic	OR (95% CI)	*p*-value
miR-210 (2^−ΔΔCt^)	4.823 (2.319–9.876)	0.02
NGAL (ng/mL)	1.005 (0.546–1.847)	NS
CysC (mg/L)	0.823 (0.446–1.483)	NS
SOFA scores (points)	3.541 (1.859–6.587)	0.03

NGAL, neutrophil gelatinase-associated lipocalin; CysC, cystatin C; SOFA, sequential organ failure assessment; NS, no significance.

## 4 Discussion

Once patients with sepsis also develop acute kidney injury, it will lead to retention of water and sodium in the body, disruption of acid–base balance, and electrolyte imbalance, thereby increasing the mortality rate of the patients (28). Therefore, early diagnosis and evaluation of sepsis-associated acute kidney injury are very important for treatment and prognosis.

CysC is mainly produced by nucleated cells in the human body, and its production rate is relatively stable. It is not affected by internal factors such as inflammatory factors, bilirubin, and triglycerides. It also has no significant correlation with general data such as age and gender (29). Since the kidneys are the only organ that can filter CysC, when the glomerular filtration function is impaired, the level of CysC in the patient’s serum will increase and continue to rise as the renal injury progresses (30). The results of this study showed that the level of CysC in patients with sepsis-associated acute kidney injury was higher than that in sepsis patients, which was consistent with previous studies (31, 32).

Scr is an important method for clinical diagnosis of kidney injury (33). However, Scr only increases when the glomerular filtration rate drops below 50%, which cannot timely diagnose kidney injury (34). BUN belongs to the nitrogen-containing compounds that seep out from the glomeruli of the kidneys and are excreted from the body (35). They are clinically used as indicators of glomerular filtration function. The results of this study demonstrated that Scr and BUN levels in patients with sepsis-associated acute kidney injury were higher than those in sepsis patients, consistent with previous literature (36).

NGAL is a 25 kD secretory glycoprotein, a member of the lipocalin superfamily, expressed in renal tubular epithelial cells, neutrophils, and vascular endothelial cells (37). Under normal physiological conditions, NGAL expression is low in renal tissues. When tissue ischemia or toxins damage renal tubules, renal tubular epithelial cells can secrete NGAL within 2 h after injury, inducing neutrophil apoptosis in the tubule interstitium, thereby reducing kidney injury (38). At the same time, high expression of NGAL can also enable renal tubular epithelial cells to repair and protect renal function (39). As previously reported, NGAL levels in the blood, renal cortical tubules, and urine were elevated in patients with acute kidney injury (38). Consistently, this research demonstrated that NGAL levels in patients with sepsis-associated acute kidney injury were elevated relative to those of sepsis patients.

The SOFA score, as an internationally recognized tool for assessing organ dysfunction in critically ill patients, is fundamentally valuable for comprehensively quantifying the severity of the condition through indicators of multiple organ systems (such as circulation, respiration, coagulation, liver, nervous system, and kidney functions) (40). It has been widely used for risk stratification and prognosis assessment in patients with sepsis (41). This study further validated its benchmark role in patients with sepsis-associated acute kidney injury—the SOFA score of such patients was significantly higher than that of patients with sepsis alone.

miRNAs are widely found in tissues and body fluids. In recent years, studies have found abnormal expression of miRNA in patients with inflammation and infection (42). Compared with traditional protein markers, miRNA is small in size, stable in structure, rarely occurs in post-processing, and so on (43). Besides, miRNAs in blood are easy to obtain and less traumatic to patients, making them readily detectable in clinical practice (44). Therefore, from the perspective of biological characteristics, circulating miRNA is suitable as a molecular marker of sepsis (45). Current studies have discovered that a variety of miRNAs are related to the development of sepsis. Of note, miR-210 is associated with the incidence of sepsis (46). Similarly, our study found that miR-210 levels in patients with sepsis-associated acute kidney injury were elevated relative to those of sepsis patients. This further highlights the potential significance of miR-210 in the diagnosis and prognosis assessment of sepsis and its related complications. miR-210 plays a significant role in the occurrence, development, and prognosis of sepsis-associated acute kidney injury through various biological mechanisms, such as participating in the cellular hypoxia response, regulating immune responses, influencing angiogenesis, and being associated with renal cell apoptosis and autophagy (22, 46, 47). These potential biological mechanisms provide a theoretical basis for using miR-210 as a diagnostic and prognostic marker for sepsis-associated acute kidney injury. However, the specific molecular regulatory network and signaling pathways still require further in-depth research to better reveal the mechanism of miR-210 in the disease and provide new targets and strategies for clinical diagnosis and treatment.

In addition, to further verify the clinical significance of miR-210, Scr, NGAL, BUN, CysC, and SOFA scores, we verified their diagnostic efficacy, and the ROC curve confirmed that miR-210, Scr, NGAL, BUN, CysC, and SOFA scores had good sensitivity and specificity. More importantly, miR-210 showed promise as a diagnostic value in distinguishing sepsis-associated acute kidney injury from sepsis patients and warranted validation. According to the prognosis, the patients were further divided into a death group and a survival group, and the statistical differences of clinical data between the two groups were evaluated. Both miR-210 and SOFA scores were statistically significant in univariate and multivariate analyses, indicating that miR-210 and SOFA scores were closely related to the prognosis of sepsis-associated acute kidney injury and played an important role in the diagnosis and prognosis of sepsis-associated acute kidney injury, which was consistent with previous studies (48).

## 5 Strengths and limitations

This study has several strengths. First, this study selected multiple indicators to comprehensively evaluate the diagnostic value and prognostic significance of patients with sepsis-associated acute kidney injury from different perspectives. Among them, miR-210, as a new type of biomarker, has received relatively less attention in previous studies regarding its relationship with sepsis-associated acute kidney injury. This study conducted an in-depth analysis of it, providing new perspectives and clues for research in this field. At the same time, combining traditional renal function indicators with new biomarkers helps to more comprehensively and accurately assess the renal function status and prognosis of patients. Second, during the data analysis process, a variety of statistical methods were employed, such as univariate analysis and multivariate analysis, to conduct in-depth investigations into the independent effects and interrelationships of each indicator.

There are some limitations in this study. First, the sample size of this study is relatively small, which to some extent limits the generalizability and statistical power of the research results. The small sample size may not be able to fully represent the overall characteristics of patients with sepsis-associated acute kidney injury, resulting in the stability of the results being affected when analyzing the diagnostic value and prognostic significance of miR-210 and Scr and other indicators. Second, all the patients included in this study came from the same center, which may introduce selection bias and affect the generalizability of the research results. Different medical centers may have differences in patient admission criteria, treatment procedures, and medical resources, and these factors may all influence the disease progression and prognosis of the patients. Therefore, more large-scale studies conducted by multiple centers are needed to verify the generalizability and reliability of the results of this study. Third, this study adopted a retrospective research design, which has some inherent limitations. Retrospective studies rely on existing medical records, and the completeness and accuracy of the data may be affected by various factors. Moreover, retrospective studies are difficult to control for confounding factors, and there may be some unknown or unmeasured confounding factors that interfere with the research results. Fourth, although this study made every effort to control for some known confounding factors during the analysis process, there may still be some unmeasured confounding factors that affect the research results. For instance, factors such as the patient’s genetic background, lifestyle (such as diet, exercise, and smoking), and psychological state may all be closely related to the occurrence, development, and prognosis of sepsis-associated acute kidney injury. However, these factors were not measured and included in the analysis in this study. These unmeasured confounding factors may lead to deviations in the estimation of the relationship between indicators such as miR-210 and Scr and the diagnosis and prognosis of the disease, thereby affecting the accuracy of the research conclusions. In addition, although this study verified the diagnostic value of miR-210 within the sample, it is necessary to emphasize that these results need to be prospectively validated in independent external cohorts. The current high sensitivity and specificity values should be used with caution in clinical practice, especially when applied to different populations or different subtypes of sepsis, where there may be variations in performance. We suggest that future studies adopt a multicenter, large sample size prospective design and incorporate traditional renal injury indicators for the construction of a combined diagnostic model. This multi-dimensional evaluation strategy not only improves diagnostic accuracy but also reduces the risk of misjudgment due to sample noise or individual differences caused by a single biomarker.

## 6 Conclusion

This study demonstrated that miR-210, Scr, NGAL, BUN, CysC, and SOFA scores have significant diagnostic values in patients with sepsis-associated acute kidney injury, and miR-210 and SOFA scores have good prognostic values for patients with sepsis-associated acute kidney injury.

## Data Availability

The datasets presented in this study can be found in online repositories. The names of the repository/repositories and accession number(s) can be found in the article/supplementary material.
